# Structure-Based Classification Defines the Discrete Conformational Classes Adopted by the Arenaviral GP1

**DOI:** 10.1128/JVI.01048-18

**Published:** 2018-12-10

**Authors:** Rhys Pryce, Weng M. Ng, Antra Zeltina, Yasunori Watanabe, Kamel El Omari, Armin Wagner, Thomas A. Bowden

**Affiliations:** a Division of Structural Biology, Wellcome Centre for Human Genetics, University of Oxford, Oxford, United Kingdom; b Oxford Glycobiology Institute, Department of Biochemistry, University of Oxford, Oxford, United Kingdom; c Centre for Biological Sciences and Institute of Life Sciences, University of Southampton, Southampton, United Kingdom; d Diamond Light Source, Harwell Science and Innovation Campus, Didcot, United Kingdom; University of Utah

**Keywords:** X-ray crystallography, arenavirus, glycoprotein, host cell entry, structure

## Abstract

The emergence of Old and New World arenaviruses from rodent reservoirs persistently threatens human health. The GP1 subunit of the envelope-displayed arenaviral glycoprotein spike complex (GPC) mediates host cell recognition and is an important determinant of cross-species transmission. Previous structural analyses of Old World arenaviral GP1 glycoproteins, alone and in complex with a cognate GP2 subunit, have revealed that GP1 adopts two distinct conformational states distinguished by differences in the orientations of helical regions of the molecule. Here, through comparative study of the GP1 glycoprotein architectures of Old World Loei River virus and New World Whitewater Arroyo virus, we show that these rearrangements are restricted to Old World arenaviruses and are not induced solely by the pH change that is associated with virus endosomal trafficking. Our structure-based phylogenetic analysis of arenaviral GP1s provides a blueprint for understanding the discrete structural classes adopted by these therapeutically important targets.

**IMPORTANCE** The genetically and geographically diverse group of viruses within the family *Arenaviridae* includes a number of zoonotic pathogens capable of causing fatal hemorrhagic fever. The multisubunit GPC glycoprotein spike complex displayed on the arenavirus envelope is a key determinant of species tropism and a primary target of the host humoral immune response. Here, we show that the receptor-binding GP1 subcomponent of the GPC spike from Old World but not New World arenaviruses adopts a distinct, pH-independent conformation in the absence of the cognate GP2. Our analysis provides a structure-based approach to understanding the discrete conformational classes sampled by these therapeutically important targets, informing strategies to develop arenaviral glycoprotein immunogens that resemble GPC as presented on the mature virion surface.

## Abstract (precis)

The genetically and geographically diverse group of viruses within the family *Arenaviridae* includes a number of zoonotic pathogens capable of causing fatal hemorrhagic fever. The multisubunit GPC glycoprotein spike complex displayed on the arenavirus envelope is a key determinant of species tropism and a primary target of the host humoral immune response. Here, we show that the receptor-binding GP1 subcomponent of the GPC spike from Old World but not New World arenaviruses adopts a distinct, pH-independent conformation in the absence of the cognate GP2. Our analysis provides a structure-based approach to understanding the discrete conformational classes sampled by these therapeutically important targets, informing strategies to develop arenaviral glycoprotein immunogens that resemble GPC as presented on the mature virion surface.

## INTRODUCTION

Although pathobiologically diverse, arenaviruses share a genomic structure comprising a bisegmented, ambisense RNA genome. The tripartite glycoprotein complex (GPC) is encoded by the small (S) segment of the arenaviral genome and is responsible for orchestrating host cell recognition and entry (1, 2). Maturation of the GPC precursor involves proteolytic cleavage of the polyprotein into a retained and myristoylated stable signal peptide (SSP), a GP1 attachment glycoprotein, and a membrane-anchored GP2 fusion glycoprotein (1–5). Noncovalently associated protomers of SSP-GP1-GP2 are highly glycosylated and displayed as trimers on the mature virion surface (6, 7). Over the past decade, Old World (OW) and New World (NW) arenaviral glycoproteins have been subjected to numerous structural studies (6, 8–19). These analyses have revealed that both GP1 and GP2 adopt unique α/β folds, with GP2 exhibiting structural features observed in other class I fusion proteins. Fitting of the crystal structure of the Lassa virus (LASV) GP1-GP2 ectodomain into an electron cryomicroscopy-derived reconstruction of the GPC has revealed the higher-order assembly of the glycoprotein spike in a pH-neutral prefusion state and places the globular domain of GP1 in the membrane-distal region of the spike complex (6, 19).

The specificity of GP1 for a cognate host cell receptor is a key determinant of cellular and species tropism (1, 2). While most OW arenaviruses interact with the *O*-mannose glycans presented on the extracellular receptor, α-dystroglycan (α-DG) (20), LASV is also known to recognize the C-type lectin DC-SIGN (dendritic cell-specific intercellular adhesion molecule-3-grabbing nonintegrin) (21, 22) and an endosomal receptor, LAMP1 (lysosomal-associated membrane protein 1) (23, 24). Furthermore, Lujo virus, an emergent OW arenavirus, has been shown to interact with the cell surface receptor neuropilin 2 and to require tetraspanin (CD63) during host cell entry (25). NW arenaviruses belonging to clades B and D (previously referred to as clade A/B or A/rec), on the other hand, utilize the transferrin receptor 1 (TfR1) orthologues of their respective rodent hosts (26–30), and clade C arenaviruses interact with α-DG (31). The ability of NW arenaviruses, such as Machupo virus (MACV) and Junín virus (JUNV), to also utilize human TfR1 is the principal determinant of zoonosis and pathogenicity in humans (30).

Following host cell attachment, virions are internalized, and the low-pH environment within endosomes destabilizes the prefusion arenaviral GPC, which results in release of GP1 and fusogenic rearrangements of GP2 (2, 6, 32). Structural studies of OW arenaviruses have revealed significant conformational differences in GP1 when expressed alone or in association with GP2 (16, 19). In contrast, NW arenaviral GP1s are unlikely to exhibit such structural differences, given that both neutralizing monoclonal antibodies and TfR1 recognize NW arenaviral GP1s in their GP2-free state (8, 10–13, 33).

Here, we sought to delineate the roles of detachment and acidification in determining the conformation of OW and NW arenaviral GP1s. We solved the crystal structures of the GP1 glycoproteins from Loei River virus (LORV), an Asiatic rodent-borne OW arenavirus of unknown pathogenicity in humans (34), and Whitewater Arroyo virus (WWAV), an NW arenavirus associated with spillover into human populations in North America (35). Both WWAV and LORV GP1s were solved at neutral pH (7.5 to 8.0) and acidic pH (5.6 to 5.0), permitting the first direct analysis of the effect of pH on the structure of GP1 in the absence of cognate GP2. These data reveal that isolated OW and NW arenaviral GP1s are structurally unaltered by pH change and demonstrate that only OW arenaviral GP1s form a distinct GP2-free state. On a broader level, this work allows us to define the discrete conformational classes assumed by arenaviral GP1 glycoproteins.

## RESULTS

### WWAV GP1 adopts a pH-independent conformation.

**FIG 1:**
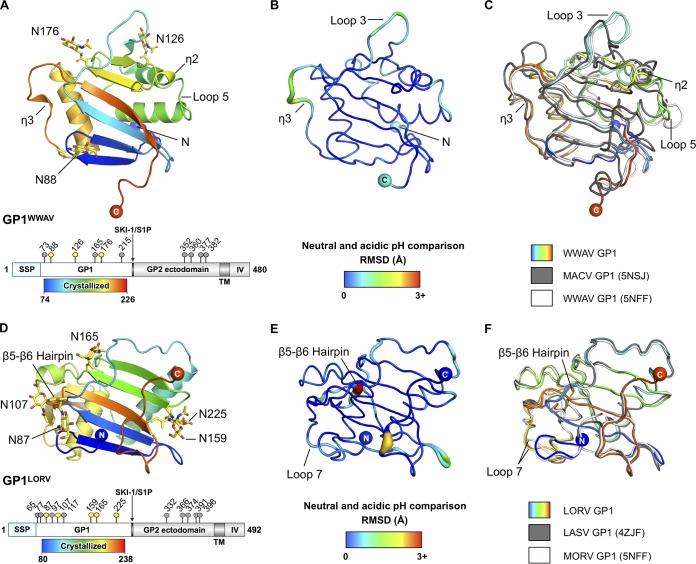
Structure and organization of the New World WWAV and Old World LORV GP1s. (A) Structure of WWAV GP1. (Top) WWAV GP1 (pH 5.6) shown as a cartoon and colored as a rainbow ramped from blue (N terminus) to red (C terminus). (Bottom) Schematic organization of WWAV GPC (generated with DOG [64]). The SSP, GP1 glycoprotein, subtilisin-like kexin protease 1–site 1 protease (SKI-1/S1P) cleavage site, GP2 glycoprotein, transmembrane region (TM), and intravirion domain (IV) are annotated. Putative N-linked glycosylation sites are labeled as pins above the schematic, with sites observed to be occupied in either the pH 7.5 or pH 5.6 crystal structure colored yellow. (B) Structural comparison of WWAV GP1 at pH 7.5 and pH 5.6. RMSDs between equivalent C-α positions are represented by both color (ramped from blue to red) and tube width (thin to thick). (C) Structure overlay of unliganded NW arenaviral GP1 structures. WWAV GP1 (pH 5.6) is shown as a rainbow, WWAV GP1 (PDB no. 5NSJ) is shown in white, and MACV GP1 (PDB no. 2WFO) is shown in gray. (D) Structure of LORV GP1. (Top) LORV GP1 (pH 5.0) is shown as a cartoon and colored as a rainbow ramped from blue (N terminus) to red (C terminus). (Bottom) Schematic organization of LORV GPC (annotated as in panel A). (E) Structural comparison of LORV GP1 at pH 5.0 and pH 8.0, with RMSDs between equivalent C-α positions represented as in panel B. (F) Structure overlay of all available OW arenaviral GP2-free GP1 structures. LORV GP1 (pH 5.0) is shown as a rainbow, LASV GP1 (PDB no. 4ZJF) is shown in white, and MORV GP1 (PDB no. 5NFF) is shown in gray.

Crystals of WWAV GP1 were generated under two conditions, buffered to pH 7.5 and 5.6, and X-ray diffraction data were collected to 2.4- and 2.0-Å resolution, respectively (Table 1). As phase determination by molecular replacement with existing arenaviral GP1 structures failed to yield a solution, the single-wavelength anomalous-dispersion (SAD) method was used for structure elucidation (Table 1). Crystallographic analysis of WWAV GP1 revealed the characteristic α/β fold that has been observed for other NW arenavirus GP1 structures, as well as in OW arenaviral GP1 glycoproteins in GP2-associated states, comprising a seven-stranded β-sheet with three α-helices positioned on the convex side of the molecule (Fig. 1A). WWAV GP1 structures determined at both neutral and acidic pHs are nearly identical (0.4-Å root mean square deviation [RMSD]) (Fig. 1B), indicating that exposure to acidic endosomal pH, and subsequent shedding of GP1 from the GPC, is unlikely to induce conformational rearrangements to the molecule.

**TABLE 1:** Crystallographic data collection and refinement statistics

Data collection statistic	Value				
	WWAV GP1^*a*^			LORV GP1^*a*^	
	pH 7.5 Cd^*b*^	pH 7.5	pH 5.6	pH 8.0	pH 5.0
Beamline	DLS I23	DLS I04	DLS I04	DLS I03	DLS I03
Wavelength (Å)	2.7552	0.9795	0.9795	0.9763	0.9763
Space group	*P*6_3_22	*P*6_3_22	*P*6_3_22	*P*4_1_2_1_2	*P*4_3_2_1_2
Cell dimensions *a*, *b*, *c* (Å)	106.9, 106.9, 74.9	106.7, 106.7, 74.8	109.0, 109.0, 70.8	60.5, 60.5, 96.4	57.3, 57.3, 113.2
α, β, γ (°)	90, 90, 120	90, 90, 120	90, 90, 120	90, 90, 90	90, 90, 90
Resolution range (Å)	75–2.99 (3.04–2.99)	29–2.43 (2.49–2.43)	55–2.08 (2.13–2.08)	39–2.51 (2.55–2.51)	40–1.98 (2.01–1.98)
*R*_merge_	0.132 (>1)	0.068 (>1)	0.132 (>1)	0.112 (>1)	0.062 (>1)
*I*/σ(*I*)	25.2 (2.0)	22.8 (1.2)	18.0 (1.6)	15.4 (1.4)	22.3 (1.5)
CC_1/2_	0.999 (0.723)	1.000 (0.638)	0.999 (0.588)	0.999 (0.522)	1.000 (0.585)
Completeness (%)	94.3 (85.0)	99.9 (100)	100 (100)	99.2 (94.9)	100 (97.9)
Multiplicity	28.3 (19.5)	18.8 (20.3)	19.2 (19.9)	11.8 (3.7)	15.2 (5.5)
Anomalous multiplicity	15.9 (4.8)				
Refinement statistics					
Resolution (Å)		29–2.43	55–2.08	39–2.51	33–1.98
No. of reflections		17,895	15,403	6,480	13,746
*R*_work_/*R*_free_		0.220/0.252	0.177/0.207	0.208/0.238	0.189/0.237
No. of atoms					
Protein		1,160	1,170	1,260	1,268
Ligand/ion		5	0	6	6
Water		0	83	14	70
Glycan		42	42	56	70
B factors (Å^2^)					
Protein		105.2	48.1	71.9	56.2
Ligand/ion		108.1	NA	103.1	87.2
Water		NA	53.3	69.0	61.9
Glycan		140.1	75.0	103.2	87.1
Ramachandran (%)					
Favored		97.9	96.6	96.8	98.7
Allowed		2.1	3.4	3.2	1.3
Outlier		0	0	0	0
RMSD					
Bond length (Å)		0.002	0.007	0.002	0.010
Bond angle (°)		0.55	0.86	0.42	0.96

a The value for the highest-resolution shell is shown in parentheses. NA, not applicable.

b Cd denotes the cadmium-derived anomalous scattering data set used for phase determination.

Another structure of WWAV GP1 has recently been reported by Shimon et al. (18). Overlay analysis revealed that while the independently reported WWAV GP1 structures were essentially identical and exhibited an RMSD of 0.9 Å, minor structural differences were observed in loop 5 of the glycoprotein, indicating inherent flexibility in the region or a requirement for the quaternary architecture of the GPC for stabilization (Fig. 1C).

Despite utilizing a common receptor, WWAV exhibits a low level of sequence conservation with other NW arenaviral GP1 glycoproteins with known structures (e.g., 24% and 25% identity to clade B JUNV and MACV, respectively), reflective of its classification as a clade D NW arenavirus. Consistent with a low level of sequence conservation with JUNV and MACV, WWAV GP1 exhibits significant structural variation throughout the α/β fold (2.3- to 2.5-Å RMSD) (Fig. 1C). These structural differences may have arisen from coevolution with individual rodent TfR1 orthologues (36) combined with immunological pressure from the host.

### LORV GP1 adopts a pH-independent GP2-free conformation.

LORV GP1 crystals were generated under both neutral (pH 8.0) and acidic (pH 5.0) conditions, and X-ray diffraction data were collected to 2.5- and 2.0-Å resolution, respectively. Neutral- and acidic-pH-derived LORV GP1 structures were solved by molecular replacement, using the crystal structure of GP2-free LASV GP1 as a search model (16) (Table 1). Structural overlay analysis revealed that the two LORV GP1 structures are highly similar (0.7-Å RMSD), indicating that pH does not modulate the conformation of isolated GP1 (Fig. 1D and E).

In contrast to the structural differences observed between WWAV GP1 and other NW arenaviral GP1 glycoproteins (Fig. 1C), LORV GP1 exhibits a high level of structural conservation with other OW arenaviral GP1 glycoproteins in GP2-free states (Fig. 1F), where superimposition of LORV GP1 with LASV GP1 and Morogoro virus (MORV) GP1 resulted in a remarkably low overall RMSD (approximately 0.8 Å and 0.7 Å, respectively). Overlay of LORV GP1 with LASV GP1 in the GP2-associated state, on the other hand, revealed substantial differences between the molecules. Indeed, consistent with previous comparisons of LASV GP1 structures (37), more than 50% of C-α atoms failed to align upon overlay of the two structures, suggesting that GP2 plays a role in stabilizing GP1 in the GP2-associated conformation likely to exist on the mature virion.

**FIG 2:**
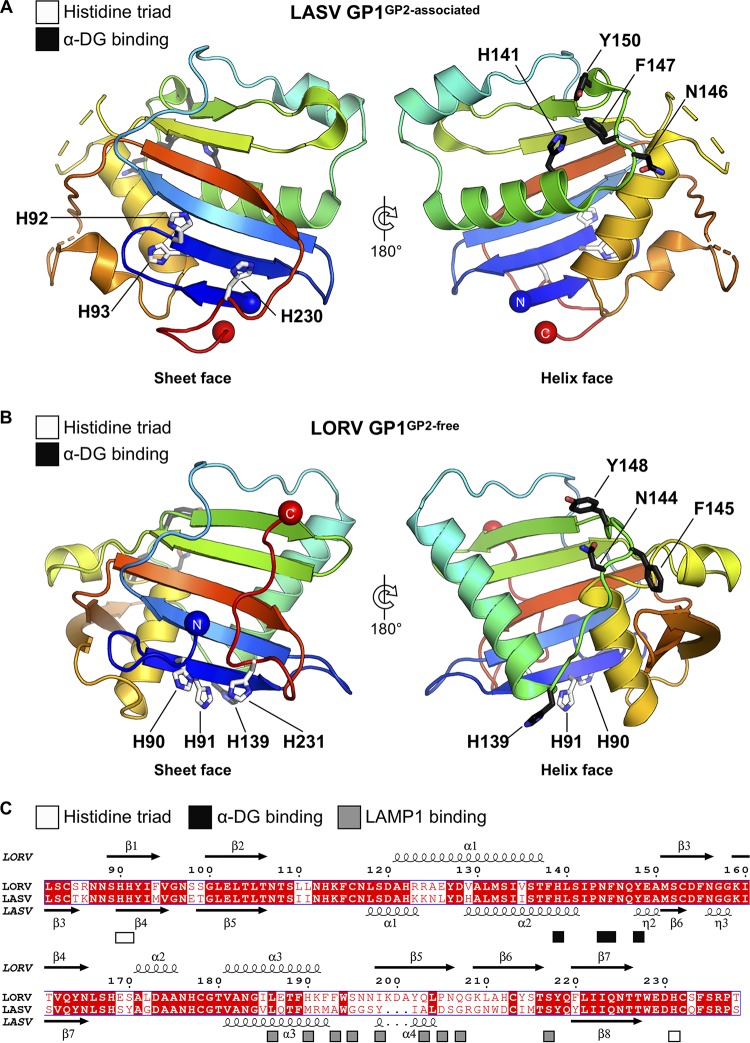
Comparison of the GP2-associated state of LASV GP1 and the GP2-free state of LORV GP1. (A) Crystal structure of LASV GP1 in the GP2-associated state (PDB no. 5VK2) is shown in cartoon representation colored as a rainbow from the N terminus (blue) to the C terminus (red). The structure of LASV GP1 was truncated to display only residues L84 to S237 to aid comparison with LORV GP1. Unresolved regions of the polypeptide are displayed as dashed lines. Residues implicated in α-DG binding are displayed as black sticks, and residues comprising the pH-sensing histidine triad are shown as white sticks, with constituent nitrogen and oxygen atoms colored blue and red, respectively. Highlighted residues are labeled according to the LASV GP1 numbering. (B) Crystal structure of LORV GP1 (pH 5.0) in the GP2-free state is shown in cartoon representation and presented as in panel A. Highlighted residues are labeled according to LORV GP1 numbering. (C) Sequence alignment of the structurally resolved region of LORV GP1 with LASV GP1. Identical residues are shaded in red, and nonidentical residues are colored red. Residues constituting the histidine triad (16) are annotated beneath the sequence with white boxes. Residues critical for α-DG (38) and LAMP1 (17) binding are annotated beneath the sequence with black and gray boxes, respectively. Secondary-structure elements of the LORV GP1 and LASV GP1 crystal structures are annotated above and below the alignment, respectively, with helices shown as coils and β-strands as arrows. LASV GP1 secondary-structure labels were assigned based on the GP2-associated LASV GP1 structure (PDB no. 5VK2). Sequences are labeled according to LORV GP1 numbering. Sequence alignments were determined with MultAlin (65) and plotted with ESPript (66).

We note that residues known to interact with α-DG (38) in LASV GP1 are fully conserved in LORV GP1, indicative of shared receptor usage. Interestingly, structure-based mapping revealed that these residues are spatially dispersed on LORV GP1. For example, in contrast to the close spatial association of critical binding residues (H141, N146, F147, and Y150) in the GP2-associated state of LASV GP1 (Fig. 2A), H139 from LORV GP1 is displaced by more than 15 Å from the nearest other predicted binding site residue (N144) (Fig. 2B). Similar to previous structural analyses of LASV GP1 and MORV GP1 (16, 17), the spatial delocalization of these receptor-binding residues in LORV GP1 is consistent with the structure constituting an α-DG binding-incompetent conformation formed following detachment from GP2 during host cell entry.

Additional mapping analysis revealed that residues expected to be crucial for LAMP1 recognition in LASV GP1 (17) were not well conserved in LORV GP1 (Fig. 2C), indicating that LORV likely undergoes a LAMP1-independent host cell entry pathway. Interestingly, however, we note that the presence and location of a histidine triad reported to function as a pH sensor for LAMP1 binding on LASV GP1 (H92, H93, and H230) (16) are conserved in LORV GP1 (H90, H91, and H231) (Fig. 2). In line with previous studies of MORV GP1 (17), we suggest that the conservation of this multihistidine motif among OW arenaviruses indicates the existence of a possible pH-sensing functionality that is independent of LAMP1 recognition, such as modulating GP1 detachment from the GPC.

### LORV GP1 is highly glycosylated.

**FIG 3:**
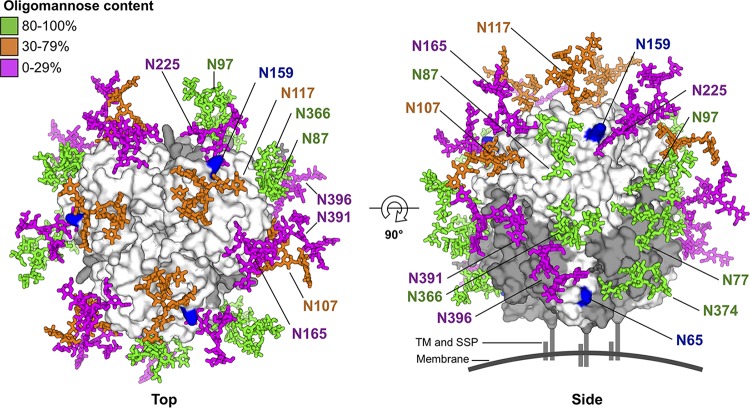
Mapping of LORV N-linked glycosylation sites onto the structure of trimeric LASV GP1-GP2. LASV GP1 and GP2 are shown as white and gray surfaces (PDB no. 5VK2), respectively. Glycans are modeled as sticks and colored according to the oligomannose content defined for the LASV GPC (7). For clarity, only glycans from a single GP1-GP2 protomer are labeled. LORV GP1 contains two additional N-linked glycosylation sites, N65 and N159, which are not found in LASV GP1 (shown as blue surfaces). A schematic representation of the viral membrane, the TM region of GP2, and the SSP is shown.

LORV GP1 encodes nine N-linked glycosylation sequons (NXT/S, where X is not P), seven of which are present in our crystallized construct. Electron density corresponding to well-ordered asparagine-linked *N*-acetylglucosamine moieties was observed at five of the seven sequons in LORV GP1 (Asn87, Asn107, Asn159, Asn165, and Asn225), and no clear density was observed at the remaining sites (Asn97 and Asn117), supportive of these sites being either disordered in the crystal or not glycosylated during protein folding. Additional glycosylation sites, Asn65 and Asn77, are located outside the boundaries of our LORV GP1 expression construct (residues 80 to 238), and mapping of these residues onto the crystal structure of the trimeric LASV GPC indicates that they are likely located in a membrane-proximal region of the glycoprotein spike (Fig. 3).

Glycosylation on the arenaviral GPC has been shown to promote evasion of the humoral immune response (39). We note that LORV GP1 contains two putative N-linked glycosylation sites (Asn65 and Asn159) that are not observed on most OW arenaviruses, including LASV. When mapped, these sites are proximal to areas that have been observed to present underprocessed, high-mannose-type glycans on the LASV GPC (7), indicating that glycosylation at Asn65 and Asn159 may contribute to an expanded glycan patch (Fig. 3). Such high glycan density on the LORV GPC suggests that LORV may also be an “evasion strong” virus (7, 40) with heightened resistance to antibody-mediated neutralization. Additionally, given the established role of high-mannose glycans in DC-SIGN-mediated entry of LASV into monocyte-derived dendritic cells (21), it is possible that the high glycan density presented on the LORV GPC may also facilitate a C-type lectin-mediated host cell entry pathway.

### Structure-based classification of arenaviral GP1 glycoproteins.

Structure-based phylogenetic analysis has been successfully utilized to demonstrate the functional and evolutionary relationships of both cellular and viral proteins (41–45). We used the Structural Homology Program (SHP) (46) to delineate the molecular features of arenaviral GP1 glycoproteins and to relate them to their functionalities and genetic lineages.

**FIG 4:**
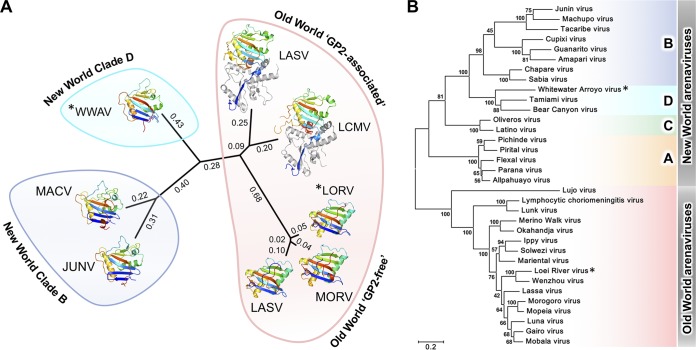
Phylogenetic analysis of arenaviral glycoproteins. (A) Structure-based phylogenetic analysis classified arenaviral GP1 glycoproteins according to genetic relatedness and structural states. Pairwise distance matrices were calculated with SHP (46) and plotted with PHYLIP (61). The LORV and WWAV GP1 structures solved in this study are annotated with asterisks. (B) Maximum-likelihood phylogeny of 34 arenaviral GPC sequences dividing the family into Old World and New World (clades A to D) groupings.

Concomitant with sequence-based phylogenetic analysis of arenaviral glycoproteins, our structure-based approach divides arenaviral GP1s according to Old and New World origins (Fig. 4). At a finer level, OW arenaviral GP1 glycoproteins bifurcate into GP2-associated and free structural states. Indeed, we observed that the structural similarity between the GP1 glycoproteins of LASV and lymphocytic choriomeningitis virus (LCMV) in their GP2-associated states was greater than that observed between the two known conformations of LASV GP1. The pronounced conformational variation of LASV GP1 structures underscores the utility of the structure-based phylogenetic approach in distinguishing discrete functional states of proteins that possess identical primary sequences.

Another striking feature of our structure-based phylogeny is that although the GP1s from WWAV, MACV, and JUNV all utilize TfR1 as a receptor, WWAV is approximately equidistant from MACV/JUNV and GP2-associated OW arenaviral GP1 structures (Fig. 4A). The marked structural variation of the NW arenaviral GP1 glycoprotein scaffold within TfR1-tropic viruses likely reflects sequence diversification in the GP1-interacting apical domain of rodent TfR1 orthologues, as well as the varied residues capable of mediating the GP1-TfR1 interaction (18).

## DISCUSSION

The arenaviral GP1 is responsible for host cell attachment and is a major determinant of cell-type and species tropism (1). In this study of WWAV GP1 and LORV GP1, we provide a blueprint for understanding the discrete structural classes formed by the arenaviral GP1 (Fig. 4). Importantly, this constitutes the first comparison of NW and OW arenaviral GP1s at both neutral and acidic pHs (Fig. 1). This investigation expands our appreciation of the structural landscape covered by arenaviral glycoproteins and provides evidence that pH change does not directly modulate the conformation of isolated GP1.

Comparison of our WWAV GP1 with other NW arenaviral GP1 structures revealed that while the NW arenaviral GP1 scaffold is structurally diverse, especially in loop regions, it adopts a single conformation that is independent of pH or the presence of ligand (Fig. 1A to C). We suggest that this conformation closely resembles that presented on the mature NW arenaviral GPC, a theory supported by previously reported crystal structures of NW arenaviral GP1-ligand complexes determined at neutral and acidic pHs, which showed that the conformation of the NW arenaviral GP1 does not change upon receptor or antibody recognition (8, 10–13), and solution state experiments, which demonstrated the ability of isolated NW arenaviral GP1s to recognize both TfR1 (8, 13, 18) and vaccine-elicited monoclonal antibodies (8, 10–13). Structural determination of an intact NW arenaviral GP1-GP2 complex will be required to confirm the equivalence of GP1 in the presence and absence of GP2.

Similar to NW WWAV GP1, structural analysis of OW LORV GP1 revealed that pH does not modulate the conformation of isolated GP1s (Fig. 1E). In contrast to WWAV GP1, however, the structure of LORV GP1 is distinct from that likely presented on the trimeric GPC (19) and equivalent to previously reported acidic pH structures of LASV GP1 (16) and MORV GP1 (17) in GP2-free states, which present α-DG-incompetent binding surfaces (Fig. 1F and Fig. 2 and 4). The biological importance of a GP2-free structural state has to date remained unresolved. Previous binding studies, for example, have shown that the formation of a GP2-free state and possession of the histidine triad are not the sole prerequisites for binding the intracellular receptor LAMP1 (17). We propose that the large structural-phylogenetic distance of this class from GP2-associated GP1s (Fig. 4) indicates the functional importance of the GP2-free state. Indeed, it is likely that GP2-free GP1 is antigenically distinct from GP2-associated GP1 and resembles the shed OW arenaviral GP1 detected in patient sera during acute LASV infection (32). The presentation of dramatically different epitopes by shed GP1, with respect to virion-displayed GP1, may thus contribute to the absence of neutralizing antibodies early in LASV infection (47).

The abundance of N-linked glycosylation on the arenaviral GPC further rationalizes the difficulty of raising an effective antibody-mediated immune response to OW arenaviruses, such as LASV (48, 49). Indeed, by analogy to human immunodeficiency virus type 1 (HIV-1) (50), N-linked glycosylation encompasses much of the trimeric LASV GPC, shielding the antigenic protein surface (7, 51). Interestingly, our mapping analysis revealed that glycan-mediated masking of the LORV GPC is likely to be even more pronounced than that of the LASV GPC (Fig. 3) and indicated the existence of arenaviruses with potentially greater glycan-mediated immune-evasive properties.

The continued threat that pathogenic arenaviruses pose to human health is exacerbated by a paucity of approved vaccines and therapeutics. We suggest that consideration of the distinct structural classes formed by arenaviral GP1 glycoproteins is of critical importance for the design of immunogens capable of eliciting neutralizing antibodies against the GPC, as displayed on the mature arenavirus surface.

## MATERIALS AND METHODS

### Protein expression and purification.

Constructs encoding the GP1 glycoprotein subunits of WWAV GP1 (residues 74 to 226; NCBI reference sequence YP001911113.1) and LORV GP1 (residues 80 to 238; GenBank accession number AHE76159.1) were PCR amplified from codon-optimized cDNA (GeneArt, Life Technologies) and cloned into the pHLsec mammalian expression vector (52).

Human embryonic kidney (HEK) 293T cells (ATCC CRL-1573) were transiently transfected with the desired protein constructs in the presence of the class 1 α-mannosidase inhibitor kifunensine (53). Cell supernatants were harvested 72 h after transfection and diafiltered against a buffer containing 10 mM Tris (pH 8.0) and 150 mM NaCl (ÄKTA Flux diafiltration system; GE Healthcare). Glycoproteins were purified by immobilized nickel affinity chromatography followed by size exclusion chromatography (SEC) using a Superdex 200 10/300 Increase column (GE Healthcare) and equilibrated in 10 mM Tris, pH 8.0, 150 mM NaCl buffer. Similar to previous solution state analyses of arenaviral GP1 glycoproteins (30, 31), both LORV GP1 and WWAV GP1 formed putative monomers in solution at both neutral and acidic pHs, consistent with the observation that the expression of arenaviral ectodomains alone is not sufficient to form the higher-order trimers observed on the virion surface. To aid crystallogenesis, LORV GP1 and WWAV GP1 were partially deglycosylated with endoglycosidase F1 (25°C for 18 h).

### Structure determination.

Crystallization experiments were performed at room temperature using the sitting-drop vapor diffusion method (54). Crystals of WWAV GP1 were obtained under two conditions: (i) 10.1 mg/ml protein, 0.2 M potassium sodium tartrate, 2 M ammonium sulfate, and 0.1 M trisodium citrate, pH 5.6, and (ii) 11 mg/ml protein, 1 M sodium acetate, 0.1 M HEPES-Na, pH 7.5, and 0.05 M cadmium sulfate. Crystals of LORV GP1 grew under two conditions: (i) 4.5 mg/ml protein, 30% (wt/vol) polyethylene glycol (PEG) 6000, and 0.1 M citrate, pH 5.0, and (ii) 5.3 mg/ml protein, 10% (wt/vol) ethanol, and 1.5 M NaCl (buffered with 10 mM Tris, pH 8.0, from the protein solution). In all instances, crystals were cryoprotected by transfer into a solution of the respective precipitant supplemented with 25% (vol/vol) glycerol prior to flash cooling in liquid nitrogen.

X-ray diffraction data were recorded at Diamond Light Source, United Kingdom. Crystal data were indexed, integrated, and scaled with XIA2 (55). The structures of LORV GP1 were solved by molecular replacement with PHASER (56), using LASV GP1 (Protein Data Bank [PDB] accession no. 4ZJF) as a search model. Phases for WWAV GP1 (pH 7.5 crystal) were obtained experimentally using the single-wavelength anomalous dispersion (SAD) method *in vacuo* at beamline I23 (57), utilizing the anomalous signal derived from uniformly bound cadmium atoms originating from the precipitant. Heavy-atom sites and an initial trace model were generated with SHELXC/D/E using the HKL2map interface (58). For all structures, iterative rounds of model building and refinement were performed using COOT (59) and PHENIX (60), respectively. Data collection and refinement statistics are presented in Table 1.

### Structure-based phylogenetic analysis.

The structures of available arenavirus GP1 glycoproteins used for phylogenetic analysis were as follows: LORV (pH 5.0), LASV (PDB accession no. 4ZJF), MORV (5NFF), LASV (5VK2), LCMV (5INE), WWAV (pH 5.6), MACV (2WFO), and JUNV (5NUZ). For 5NUZ, 5VK2, and 5INE, all the chains not comprising GP1 molecules (e.g., GP2 and antibody fragments) were removed prior to structure alignment. A pairwise evolutionary distance matrix was created using SHP (46) and displayed as an unrooted phylogenetic tree generated using PHYLIP (61).

### Phylogenetic analysis of arenavirus GPC sequences.

An evolutionary history was inferred using the maximum-likelihood method based on the model of Le and Gascuel (62). The percentage of trees in which the associated taxa clustered together is shown next to the branches. The initial tree(s) for the heuristic search was obtained automatically by applying neighbor-joining and BioNJ algorithms (67) to a matrix of pairwise distances estimated using a Jones-Taylor-Thornton (JTT) model (68) and then selecting the topology with a superior log-likelihood value. A discrete gamma distribution was used to model evolutionary rate differences among sites. The rate variation model allowed some sites to be evolutionarily invariable. The tree is drawn to scale, with branch lengths measured in the number of substitutions per site. The analysis involved 34 arenavirus GPC amino acid sequences, classified as Old World and New World. New World arenaviruses are further categorized into four clades (A, B, C, and D). All positions containing gaps and missing data were eliminated. There were a total of 426 positions in the final data set. Evolutionary analyses were conducted in MEGA7 (63).

### Accession number(s).

Coordinates and structure factors of WWAV GP1 and LORV GP1, crystallized at neutral and acidic pHs, have been deposited in the Protein Data Bank with the accession codes 6HJ4, 6HJ5, 6HJC, and 6HJ6.
